# Supplementary figures and images for: A single-nucleus transcriptomic atlas of the adult Aedes aegypti mosquito
Source: Cell. Author manuscript; Available in PMC 2026 Jan 5. (PMC12767863; doi:10.1016/j.cell.2025.10.008)

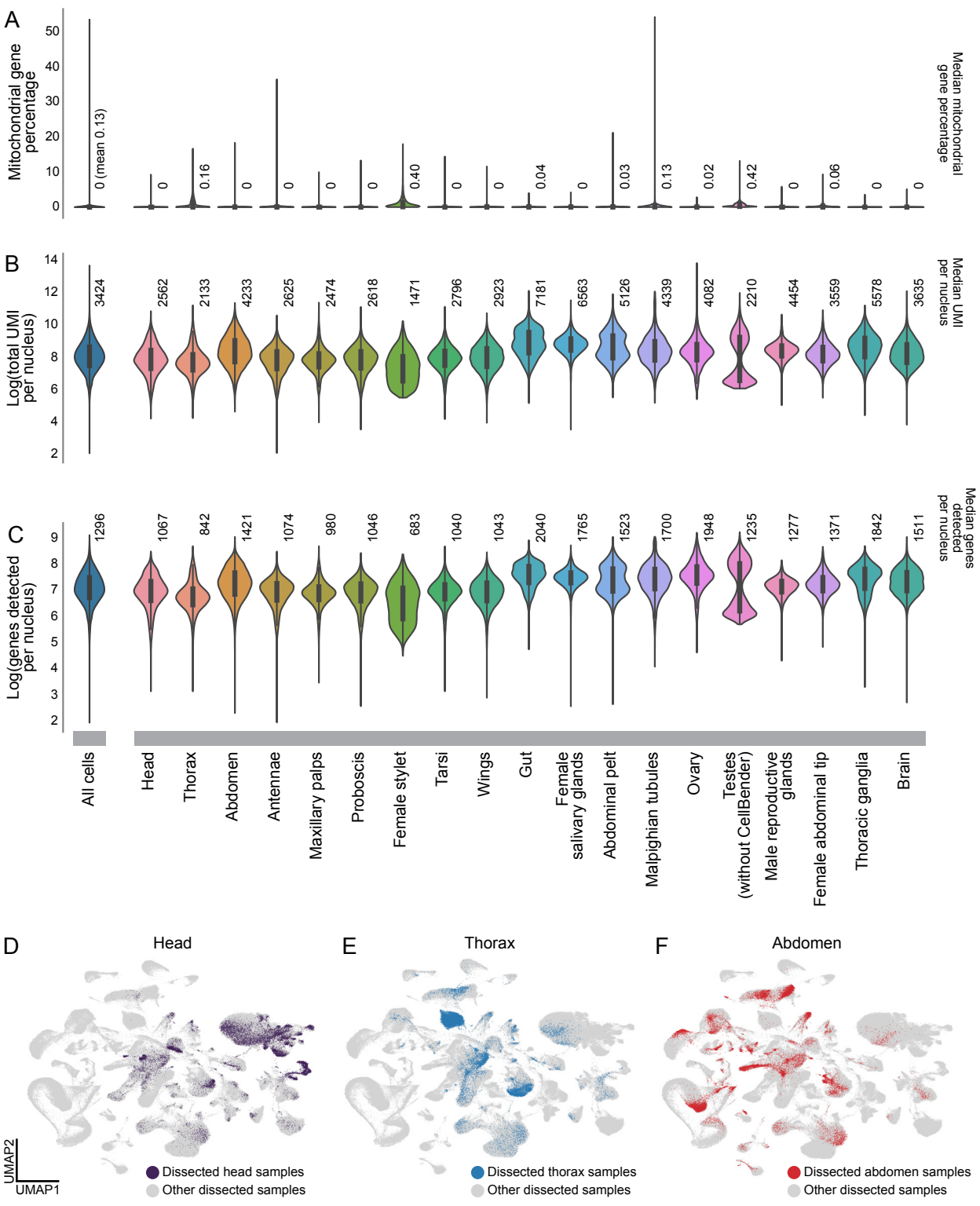

Supplement: 6 — Figure S1. Final data quality control post filtering, related to Figure 1. (A-C) Violin box plots depicting data (post-quality control filtering) mitochondrial gene percentage (A), total unique molecular identifiers (UMI) per nuclei (B), and genes detected per cell (C) for all cells (left) and for each tissue (right). Across all nuclei from all tissues, median mitochondrial gene percentage was 0.00% (mean 0.13%), median total UMI per nucleus was 3,424, and median genes per nucleus was 1,296. Annotations above each violin represent median values unless otherwise indicated. Pre-filtering gene per nucleus and UMI per nucleus metrics are available in Table S1, as well as filtering parameters. Note clusters were filtered by quality control metrics on clusters, not filtered on individual cell quality control metrics (Data S1, Table S1). Multiple samples (10x Genomics libraries) from males and females are included for each tissue. Inner boxes represent first quartile to third quartile. Box plot lines represent 1.5x interquartile range. Length of violin indicates complete range of the data, with thickness of the violin representing number of cells at each value. (D-F) UMAPs of integrated Aedes aegypti Mosquito Cell Atlas data, colored by samples representing major body parts: female and male head (D), thorax (E), and abdomen (F). [file NIHMS2117010-supplement-6.pdf]

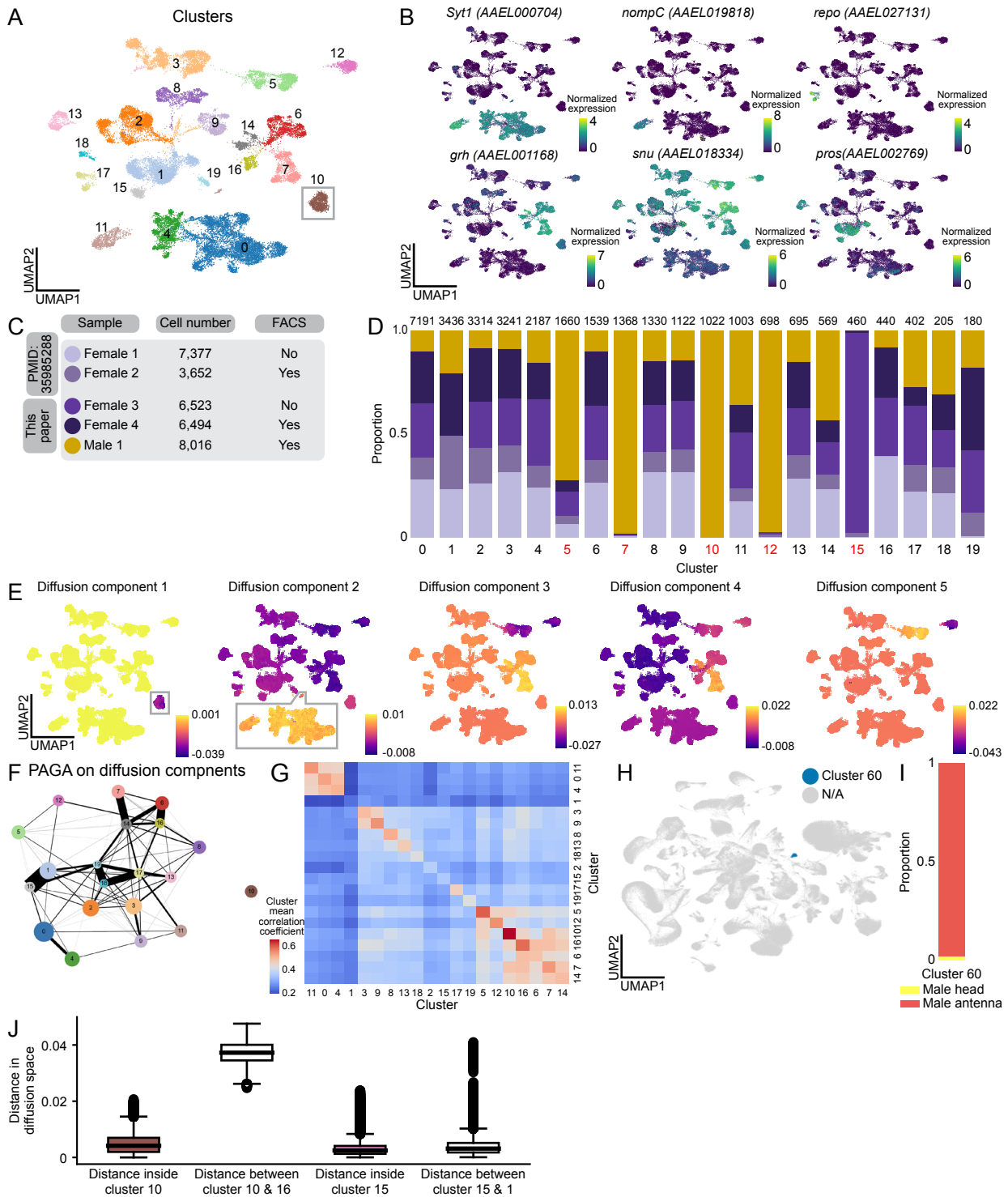

Supplement: 8 — Figure S3. Identification of male-specific ppk317 cell type in Aedes aegypti antenna, related to Figure 3. (A) UMAP of antenna nuclei clustered and numbered using the Leiden algorithm (resolution = 0.1). Cluster 10 (male-specific, ppk317-expressing cells) highlighted in grey. (B) Normalized expression of Syt1, nompC, repo (AAEL027131), grh (AAEL001168), snu (AAEL018334), and pros (AAEL002769). Normalized expression is ln([(raw count/total cell counts)*median total counts across cells]+1). (C) Number of cells in each sample (female = 4, male = 1), data source, and if each sample underwent fluorescence-activated cell sorting (FACS). (D) Stacked bar plot illustrating proportion of each sample within each cluster. Annotated information: cluster numbers (below bar plot), clusters for which over 70% originate from a single sample (red), number of cells in each cluster (above bar plot). (E) UMAPs of diffusion components 1 through 5. Diffusion component 1 (first panel) maps to cluster 10 (ppk317-expressing cells, highlighted in grey), suggesting a robust biological feature. Diffusion component 2 (second panel) maps to neurons (highlighted in grey). (F) Partition-based graph abstraction (PAGA) calculated on diffusion components in (E). All edges illustrated, no edge threshold set. (G) Correlation matrix heatmap, depicting pairwise correlation of gene expression matrices between each cluster (mean Pearson correlation coefficient). Diagonal values (cluster to itself) represents intra-cluster correlation values, which vary based on features such as cell number and transcriptome heterogeneity. (H) UMAP of integrated Aedes aegypti Mosquito Cell Atlas data with cluster 60 colored in blue (Louvain algorithm, resolution = 0.1). ppk317-expressing antennal cells belong to cluster 60, see Figure 3E. (I) Stacked bar plot indicating tissue origin of cells from cluster 60. Cluster 60 come from the male antenna and male head sample. (J) Pairwise Euclidean distances on diffusion embeddings [file NIHMS2117010-supplement-8.pdf]

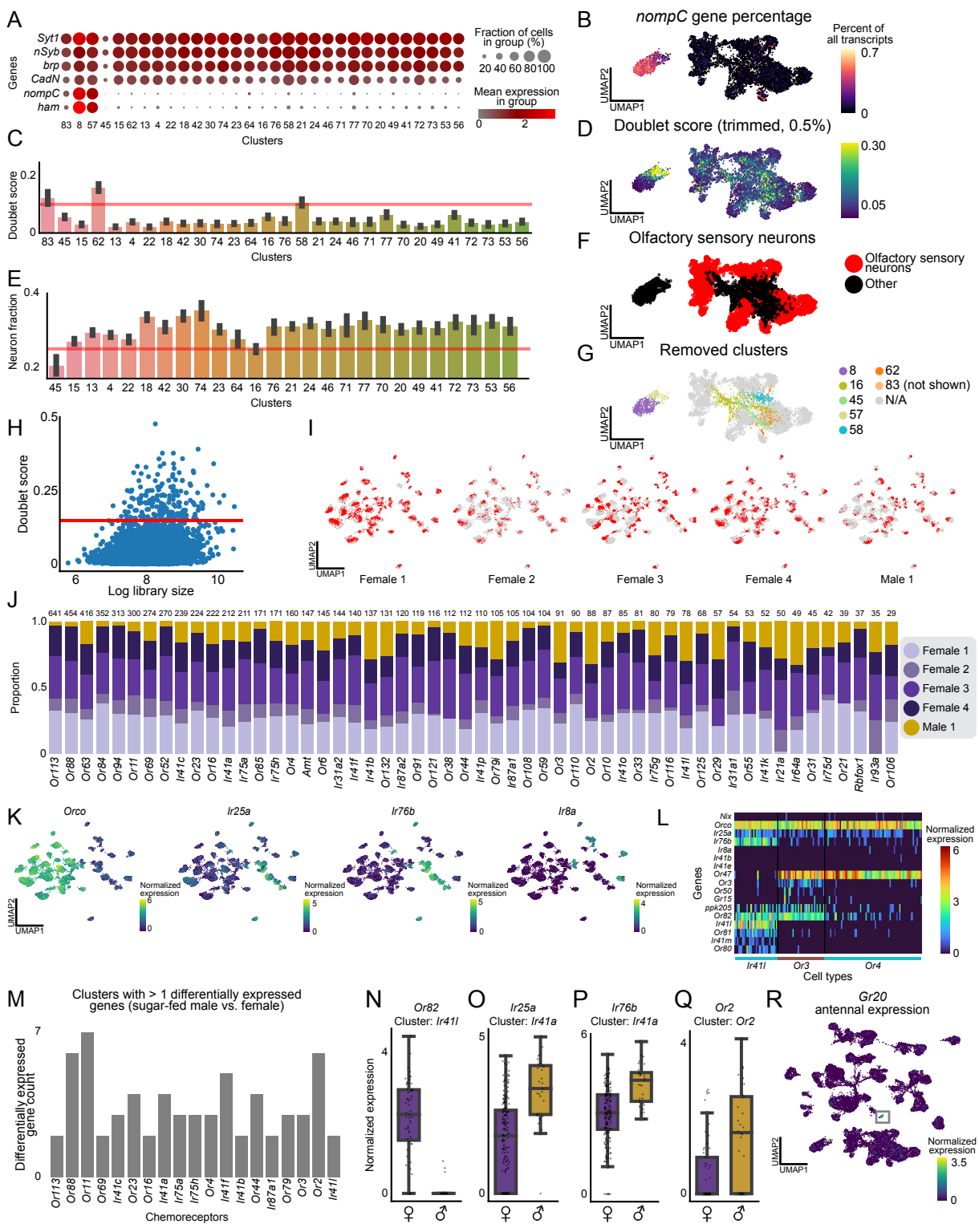

Supplement: 9 — Figure S4. Antenna chemosensory cell type expressing Orco and Ir25a is sexually dimorphic for Or82, related to Figure 4. (A) Dot plot illustrating mean normalized expression of neuronal genes set: Syt1, nSyb, brp (AAEL018153), CadN, nompC, and ham (AAEL017229). Size of dot indicated the percent of cells in each group, color indicated mean normalized expression. Normalized expression is ln([(raw count/total cell counts)*median total counts across cells]+1). (B) Fraction of total transcripts per cell of nompC in antennal neuronal nuclei. (B,D,F,G) use the same UMAP coordinates as neurons from Figure 4A. UMAPs cropped for space, cluster 83 not shown. (C) Mean doublet score across cells, with error bars indicating 95% confidence interval calculated from bootstrapping. Generated through scrublet189. Clusters with an average score above 0.15 were removed from further analysis (red line). (D) UMAP depicting doublet score. (E) Average percentage of neuronal genes in (A), with error bars indicating 95% confidence interval calculated from bootstrapping. Clusters with an average score below 0.25 were removed from further analysis (red line). (F) UMAP demonstrating which clusters (Leiden, resolution = 5) were kept for downstream analysis (red) or removed based on filtering parameters (black). (G) UMAP of neurons from antenna samples, demonstrating clusters removed from downstream analysis. (H) Log(library size) versus calculated doublet score for neurons filtered in (A-G). Cells with a score above 0.15 were removed from further analysis (red line). (I) UMAPs of antenna olfactory sensory neurons (filtered nompC-negative sensory neuron population), colored by sample. (J) Stacked bar plot illustrating proportion of each sample within each cluster in annotated nompC-negative sensory neuron population. Annotated information: cluster numbers (below bar plot), number of cells in each cluster (above bar plot). No clusters has more than 70% of cells from a single sample. (K) Normalized [file NIHMS2117010-supplement-9.pdf]

**A** Proboscis neuron gene percentage

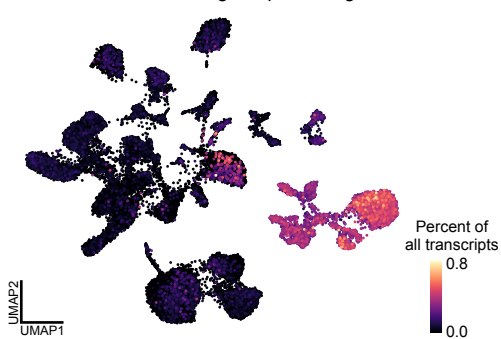

**B** Proboscis sensory neurons by sample

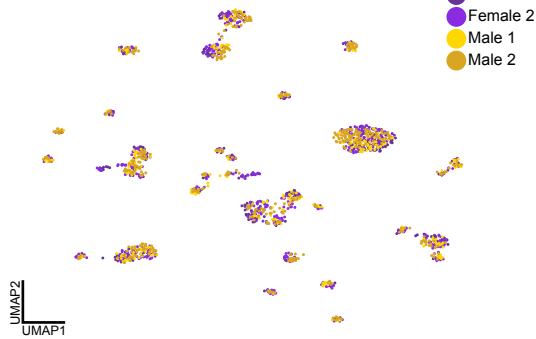

**C**

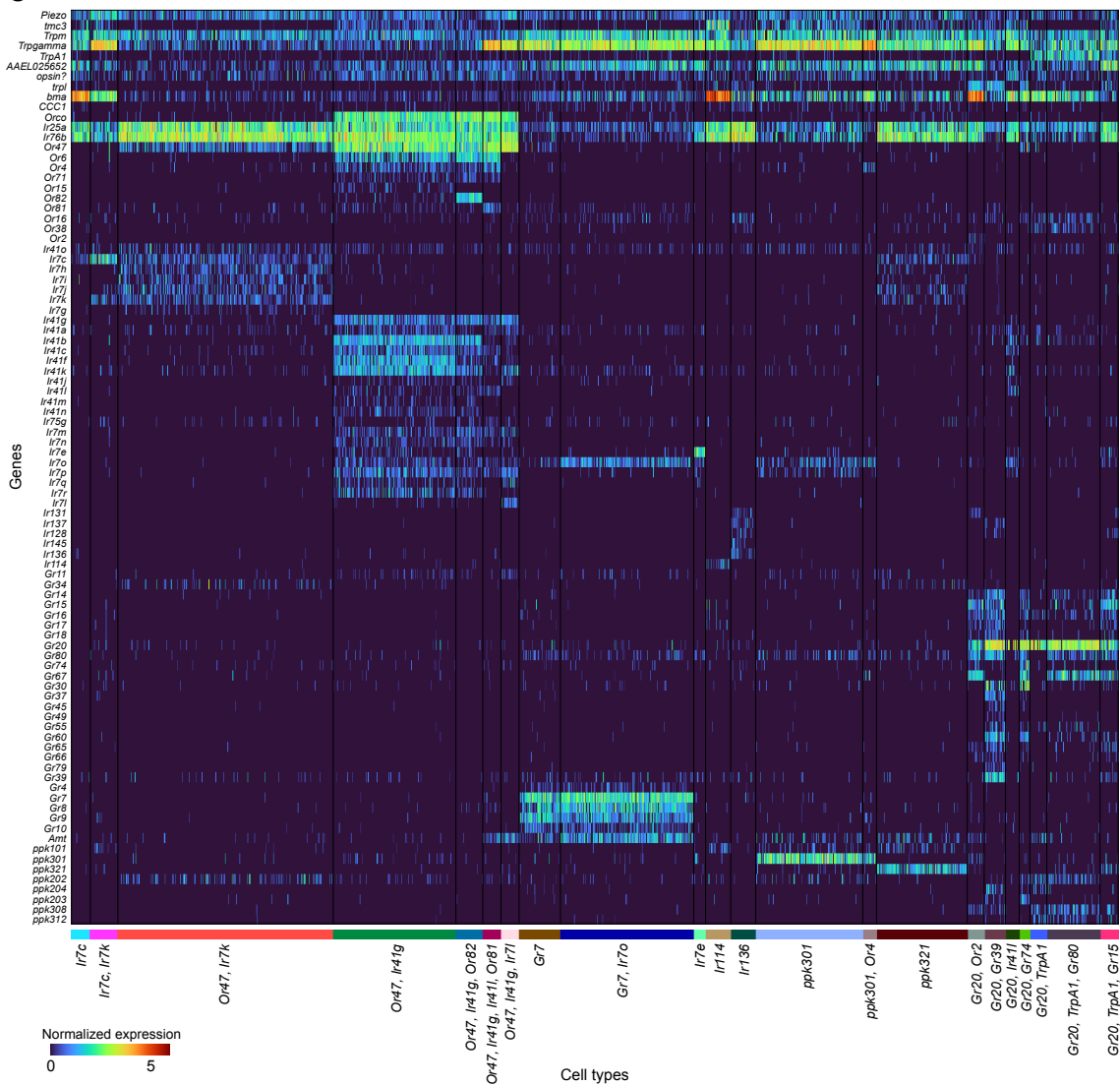

Supplement: 11 — Figure S6. Proboscis sensory gene analysis, related to Figures 4 and 5. (A) UMAP of proboscis cells illustrating fraction of total transcripts per cell of neuronal genes set: Syt1, brp, nSyb, CadN. nompC-negative cells highlighted (grey box). For nompC gene percentage, see Data S4. (B) UMAP of reclustered proboscis nompC-negative sensory cells colored by sample (female = 2, male = 2). (C) Heatmap of cells from all annotated clusters. Sensory genes are indicated in rows and cells indicated in columns. Selected genes are indicated in rows, cells in columns, with cell type annotations below. Heatmap colors represent normalized expression. Normalized expression is ln([(raw count/total cell counts)*median total counts across cells]+1). [file NIHMS2117010-supplement-11.pdf]

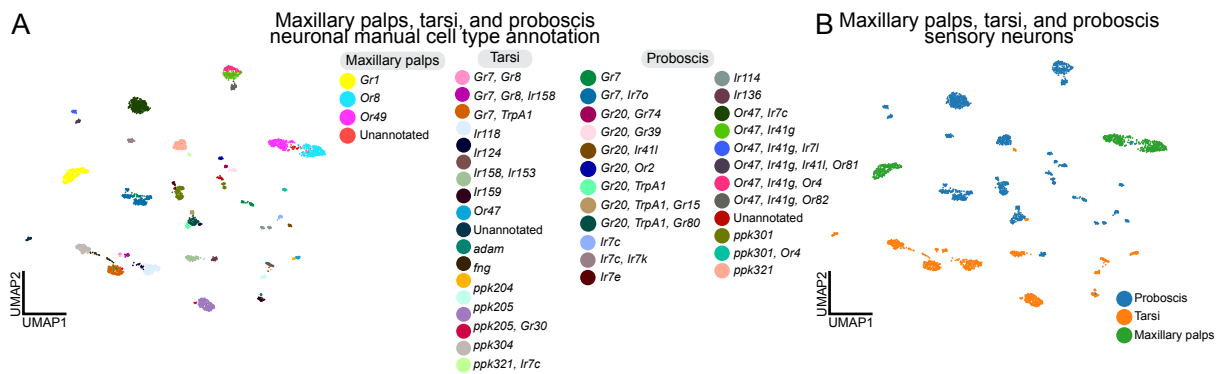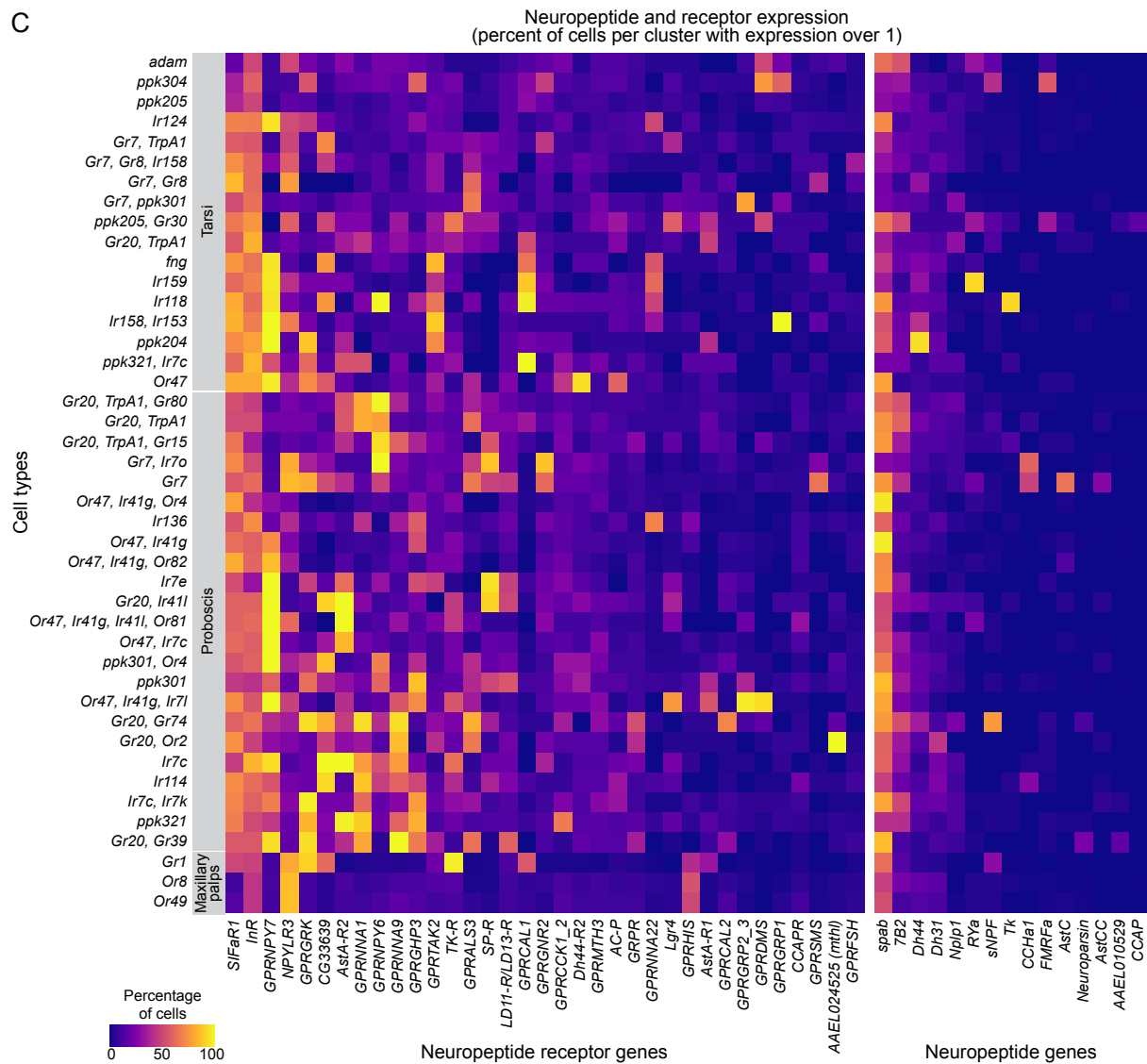

Supplement: 12 — Figure S7. Neuropeptide receptor and synthesis gene analysis, related to Figures 4 and 5. (A-B) UMAP of nompC-negative sensory neurons from maxillary palps, tarsi and proboscis samples, colored by manual cell type annotation as listed in legend at the right of the figure panel (A) and original tissue (B). (C) Heatmap of expression of neuropeptide receptor genes (left) and neuropeptide synthesis genes (right) within annotated nompC-negative sensory neurons in the maxillary palps, tarsi and proboscis. Color scale indicates percentage of cells expressing a gene above threshold (normalized expression value of 1). Sensory genes are indicated in columns and annotated cell types indicated in rows. Genes were included if they were expressed above threshold in over 20% of cells in at least one cell type. Genes filtered from lists in Table S1. Normalized expression is ln([(raw count/total cell counts)*median total counts across cells]+1). [file NIHMS2117010-supplement-12.pdf]

## A

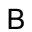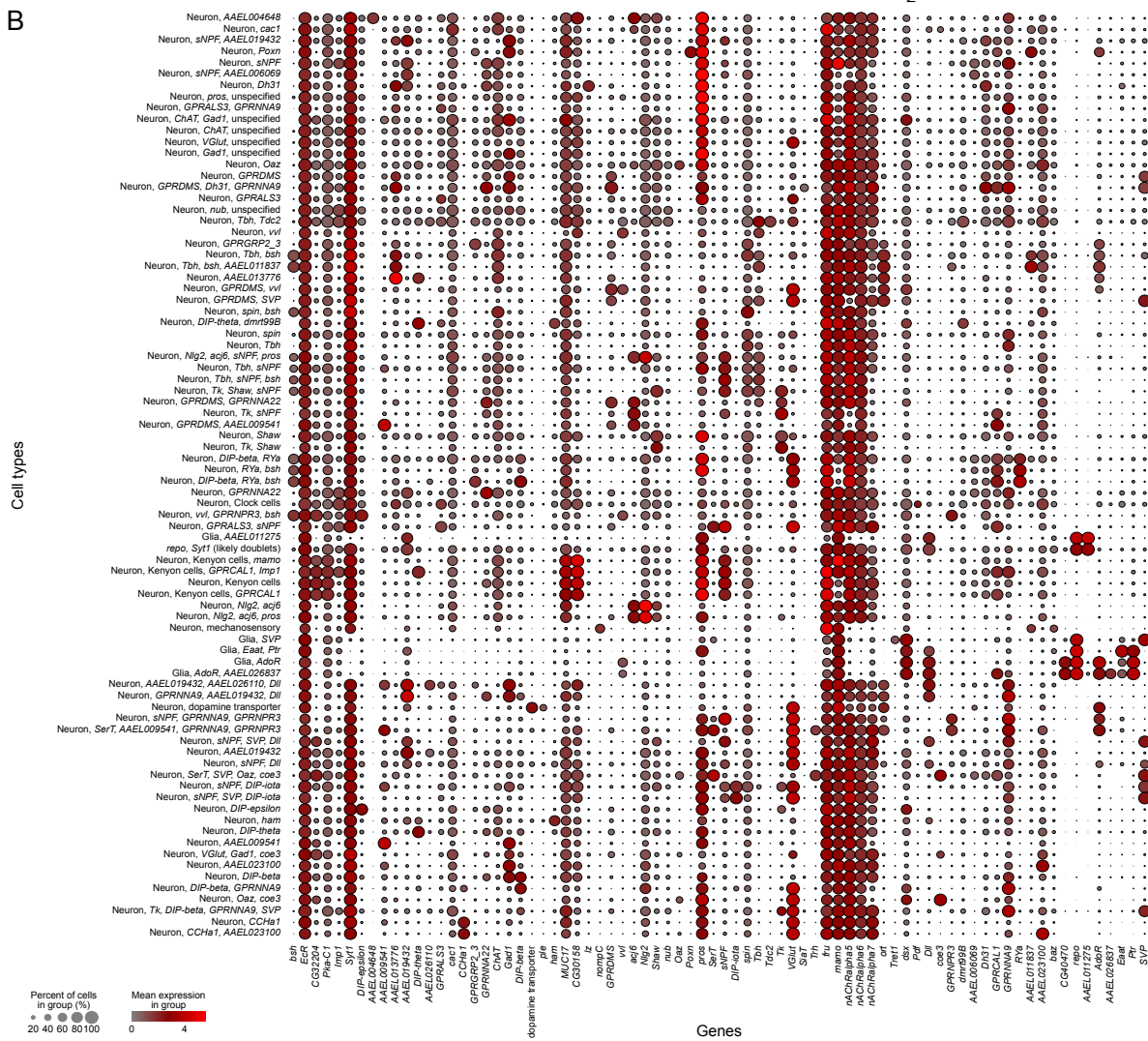

Supplement: 13 — Figure S8. Brain Cell types, related to Figures 6 and 7. (A) Stacked bar plot illustrating proportion of each sample within each cluster for brain nuclei cell types. Annotated information: cluster numbers (below bar plot), clusters for which over 70% originate from a single sample (red), number of cells in each cluster (above bar plot). (B) Dot plot illustrating mean normalized expression of gene markers. Color scale indicates mean normalized expression of gene within cell type, size of dot indicates percent of cells expressing gene within the group. See Table S1 for gene IDs and annotation thresholds. [file NIHMS2117010-supplement-13.pdf]

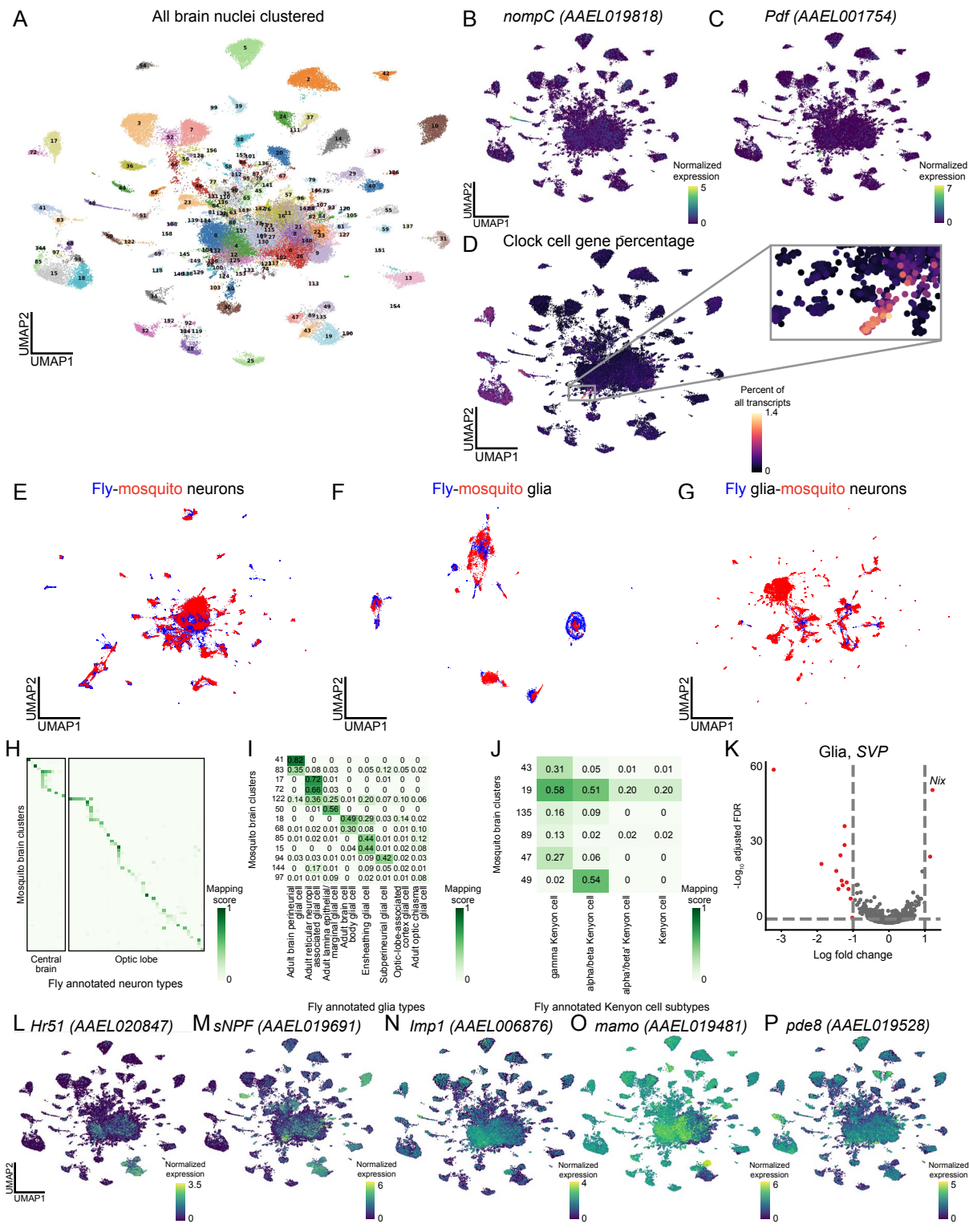

Supplement: 14 — Figure S9. SAMap analysis and identification of clock cells and kenyon cells, related to Figure 6. (A) UMAP of brain nuclei clustered using the Leiden algorithm (resolution = 5). (B-C) Normalized expression of nompC (B) and Pdf (AAEL001754) (C). Normalized expression is ln([(raw count/total cell counts)*median total counts across cells]+1). (D) Fraction of total transcripts per cell of 10 putative clock cell gene markers (Table S1). Cluster with high expression highlighted in grey box, enlarged in inset. (E-G) UMAP of manifold integration of snRNA-seq data from Aedes aegypti mosquito brain and published Drosophila melanogaster fly head18. Plots show integration of fly neurons with mosquito neurons (E), fly glia with mosquito glia (F), and, as a control, fly glia with mosquito neuron (G). Alignment scores are 0.64, 0.64 and 0.47, respectively. (H-J) Correlation matrices of mapping scores between Drosophila melanogaster head annotations and Aedes aegypti of clusters (Leiden, resolution = 5) for neuronal cell types (H), glial cell types (I), and Kenyon cell subtypes (J). For all numerical values, see Table S3. (K) Volcano plot of differentially expressed genes between male and female cells in the SVP glia (AAEL002765) cluster. Significant genes (red) had |log fold change| >1, false discovery rate <0.05, determined by MAST on normalized expression (Table S2). Male biased genes on right, indicated by Nix (AAEL022912). (L-P) Normalized gene expression of Hr51 (AAEL020847) (L), sNPF (AAEL019691) (M), Imp1 (AAEL006876) (N), mamo (AAEL019481) (O), Pde8 (AAEL019528) (P). [file NIHMS2117010-supplement-14.pdf]

**A** *HR3* (AAEL009588)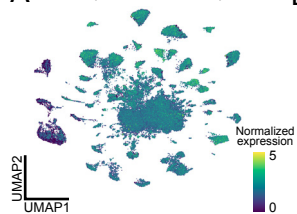**B** *E75* (AAEL007397)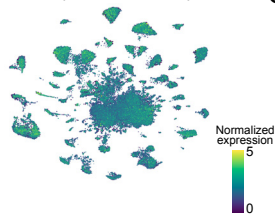**C** *EcR* (AAEL019431)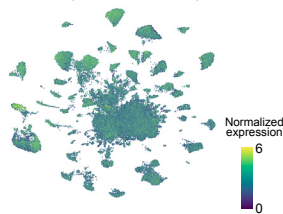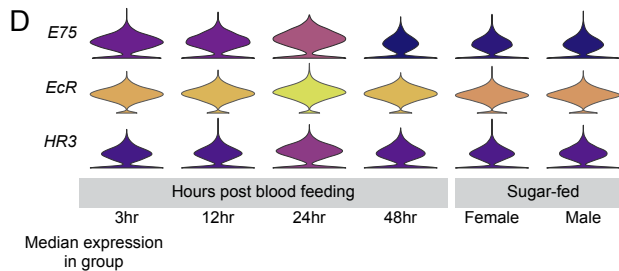**E** *EcR* (AAEL019431)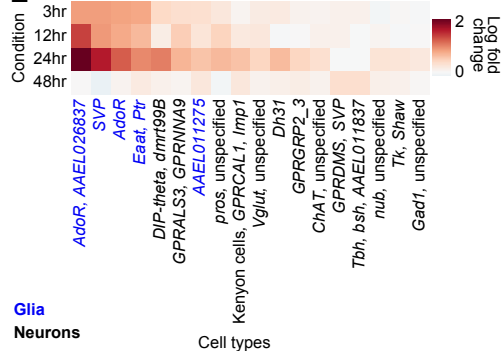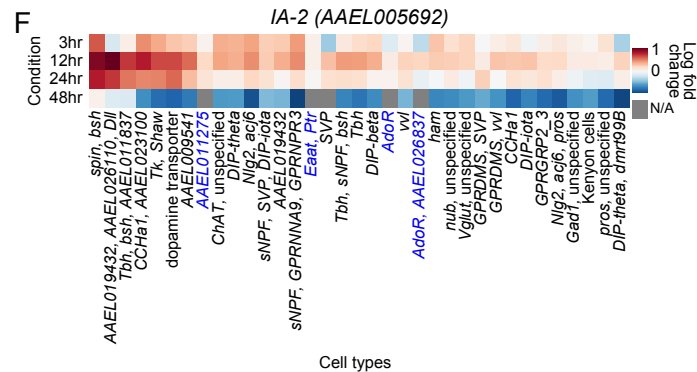**G** *dsx* (AAEL009114)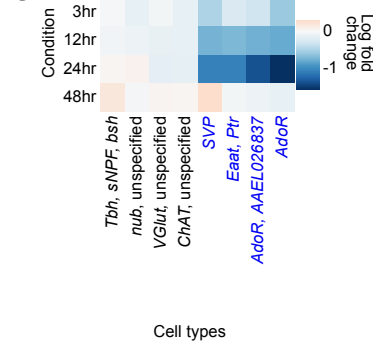**H** *fru* (AAEL024283)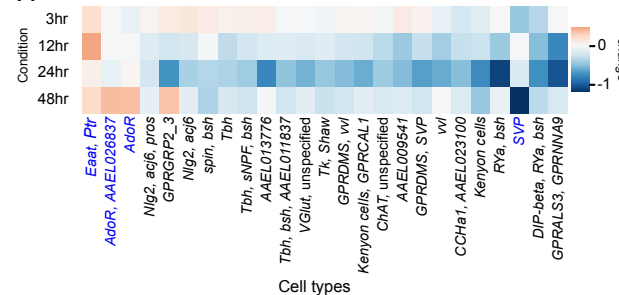**I** *ITP* (AAEL019725)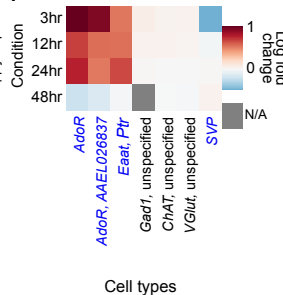**J** *PER* (AAEL008141)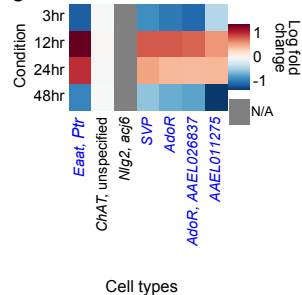

Supplement: 15 — Figure S10. Blood feeding changes in brain, related to Figure 7. (A-C) Normalized gene expression UMAP of E75 (A), EcR (AAEL019431) (B) and HR3 (C) in all brain nuclei. Normalized expression is ln([(raw count/total cell counts)*median total counts across cells]+1). (D) Violin plot of gene expression of E75, EcR and HR3 across all brain nuclei in each timepoint. Length of violin indicates complete range of the data, with thickness of the violin representing number of cells at each value. (E-J) Heatmaps of log fold change of gene expression, grouped by annotated cell type, between corresponding cells collected in each blood-feeding timepoint compared to sugar-fed female brain. Genes shown: EcR (E), IA-2 (AAEL005692) (F), dsx (AAEL009114), (G), fru (AAEL024283) (H), ITP (AAEL019725) (I), and PER (AAEL008141) (J). Log fold change is determined by MAST on normalized expression. Cell types are sorted by the total log fold change across all timepoints and colored as glia (blue) or neurons (black). Cell types included have over 10 cells in each timepoint, and at least one timepoint where change from sugar-fed condition had a false discovery rate <0.05. Grey boxes indicate log fold change data is not available, due to zero expression within cell type at the specified timepoint (or the sugar-fed condition for PER in Nlg2, acj6 cells). [file NIHMS2117010-supplement-15.pdf]
