# Supplementary material for: A single-nucleus transcriptomic atlas of the adult Aedes aegypti mosquito
Source: Cell. Author manuscript; Available in PMC 2026 Jan 5. (PMC12767863; doi:10.1016/j.cell.2025.10.008)
Supplement: 7 — Figure S2. Integrated Aedes aegypti Mosquito Cell Atlas tissues and data, related to Figure 1. (A-B) UMAPs of integrated Aedes aegypti Mosquito Cell Atlas data colored by clustering (Louvain algorithm, resolution = 1) (A) and major cell types based on manual annotation in Figure 1F (B). (C) Dot plot of integrated Aedes aegypti Mosquito Cell Atlas annotations and gene markers. Color scale indicates mean normalized expression of gene within cell type, size of dot indicates percent of cells expressing gene within the group. See Table S1 for gene IDs and annotation thresholds. [file NIHMS2117010-supplement-7.pdf]

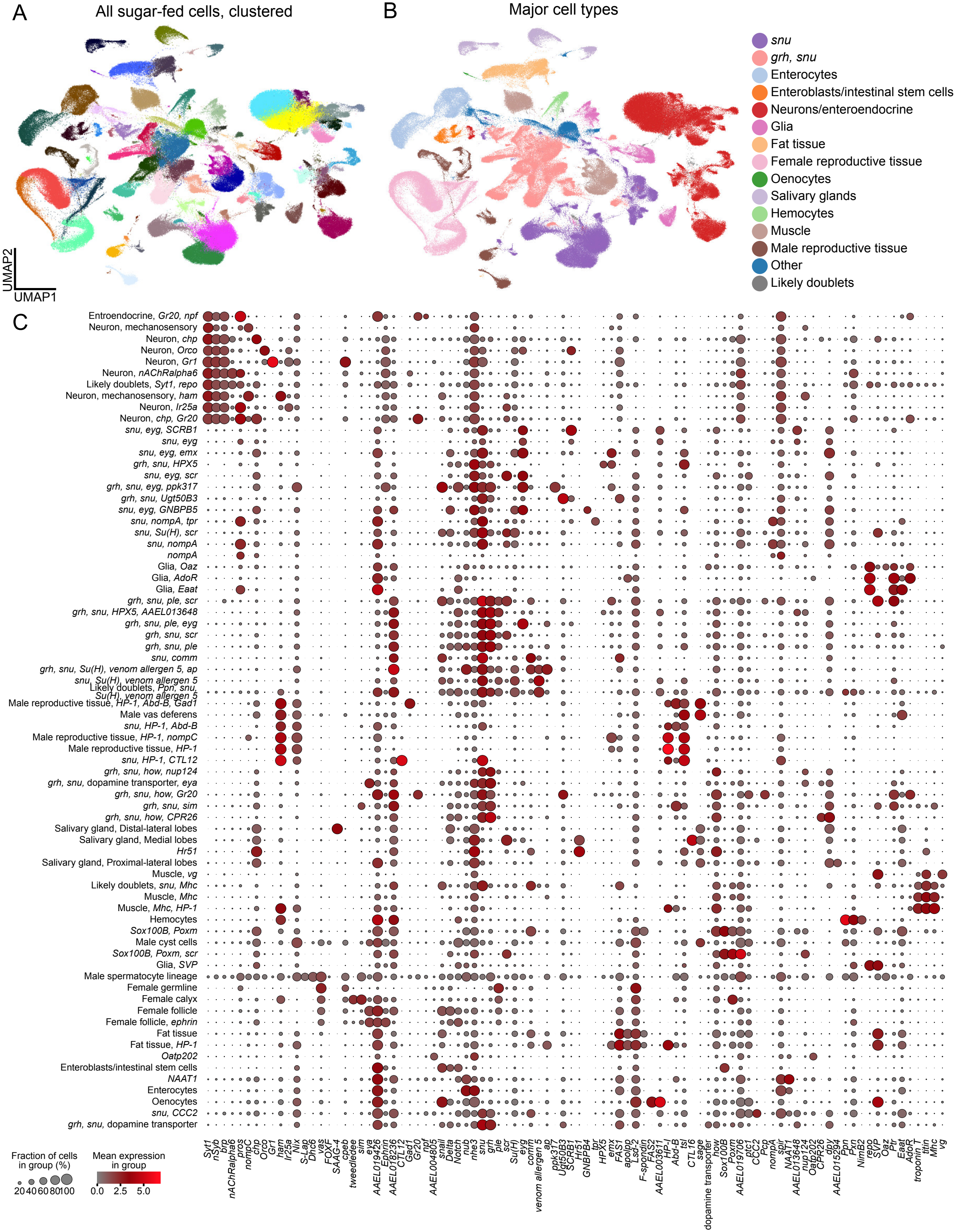

A
All sugar-fed cells, clustered
UMAP2
UMAP1
B
Major cell types
snu
grh, snu
Enterocytes
Enteroblasts/intestinal stem cells
Neurons/enteroendocrine
Glia
Fat tissue
Female reproductive tissue
Oenocytes
Salivary glands
Hemocytes
Muscle
Male reproductive tissue
Other
Likely doublets
C
Entroendocrine, Gr20, npf
Neuron, mechanosensory
Neuron, chp
Neuron, Orco
Neuron, Gr1
Neuron, nAChRalpha6
Likely doublets, Syt1, repo
Neuron, mechanosensory, ham
Neuron, Ir25a
Neuron, chp, Gr20
snu, eyg, SCRB1
snu, eyg
snu, eyg, emx
grh, snu, HPX5
snu, eyg, scr
grh, snu, eyg, ppk317
grh, snu, Ugt50B3
snu, eyg, GNBPB5
snu, nompA, tpr
snu, Su(H), scr
snu, nompA
nompA
Glia, Oaz
Glia, AdoR
Glia, Eaat
grh, snu, ple, scr
grh, snu, HPX5, AAEL013648
grh, snu, ple, eyg
grh, snu, scr
grh, snu, ple
snu, comm
grh, snu, Su(H), venom allergen 5, ap
snu, Su(H), venom allergen 5
Likely doublets, Ppn, snu, Su(H), venom allergen 5
Male reproductive tissue, HP-1, Abd-B, Gad1
Male vas deferens
snu, HP-1, Abd-B
Male reproductive tissue, HP-1, nompC
Male reproductive tissue, HP-1
snu, HP-1, CTL12
grh, snu, how, nup124
grh, snu, dopamine transporter, eya
grh, snu, how, Gr20
grh, snu, sim
grh, snu, how, CPR26
Salivary gland, Distal-lateral lobes
Salivary gland, Medial lobes
Hr51
Salivary gland, Proximal-lateral lobes
Muscle, vg
Likely doublets, snu, Mhc
Muscle, Mhc
Muscle, Mhc, HP-1
Hemocytes
Sox100B, Poxm
Male cyst cells
Sox100B, Poxm, scr
Glia, SVP
Male spermatocyte lineage
Female germline
Female calyx
Female follicle
Female follicle, ephrin
Fat tissue
Fat tissue, HP-1
Oatp202
Enteroblasts/intestinal stem cells
NAAT1
Enterocytes
Oenocytes
snu, CCC2
grh, snu, dopamine transporter
Syt1
nSyb
brp
nAChRalpha6
pros
nompC
chp
Orco
Gr1
ham
Ir25a
Nix
S-Lap
Dhc6
vas
FOXF
SAAG-4
cpeb
tweedledee
sim
eya
AAEL019426
Ephrin
AAEL018236
CTL12
Gad1
Gr20
npf
AAEL004805
snail
Delta
Notch
nub
nhe3
snu
grh
ple
scr
Su(H)
eyg
comm
venom allergen 5
ap
ppk317
Ugt50B3
SCRB1
Hr51
GNBPB4
tpr
HPX5
emx
FAS1
apolpp
Lsd-2
F-spondin
FAS2
AAEL003611
HP-I
Abd-B
tsl
CTL16
sage
dopamine transporter
how
Sox100B
Poxm
AAEL019706
ptc1
CCC2
Pcp
nompA
spir
NAAT1
AAEL013648
nup124
Oatp202
CPR26
dpy
AAEL015294
Ppn
Pxn
NimB2
repo
SVP
Oaz
Ptr
Eaat
AdoR
troponin T
titin
Mhc
vg
Fraction of cells in group (%)
20 40 60 80 100
Mean expression in group
0.0 2.5 5.0
