# Supplementary material for: A single-nucleus transcriptomic atlas of the adult Aedes aegypti mosquito
Source: Cell. Author manuscript; Available in PMC 2026 Jan 5. (PMC12767863; doi:10.1016/j.cell.2025.10.008)
Supplement: 10 — Figure S5. Antenna chemosensory neuron chemoreceptor and putative transcription factor factor expression profiles, related to Figure 4. (A-B) Heatmap of all annotated antenna olfactory sensory neuron cell types for selected putative transcription factors (A) and selected sensory genes (B). Selected genes are indicated in rows, cells in columns, with cell type annotations below. Cells are grouped by annotation in Figure 4C and indicated below heatmap. Asterisks (*) on cell type annotation indicates that a group may represent a co-clustering of cells with multiple unique sensory gene expression patterns, as depicted in Figure 4C–4D. Heatmap colors represent normalized expression. Normalized expression is ln([(raw count/total cell counts)*median total counts across cells]+1). See Table S1 for gene IDs and annotation thresholds. (C) Scatterplot of summed raw counts of sensory genes within all olfactory sensory neurons within each antenna sample. Red line indicates count of 1, blue line is a count of 5. 158 out of 403 putative sensory genes (Table S1) with the highest counts shown for space (full plot available in Zenodo Supplemental Data). [file NIHMS2117010-supplement-10.pdf]

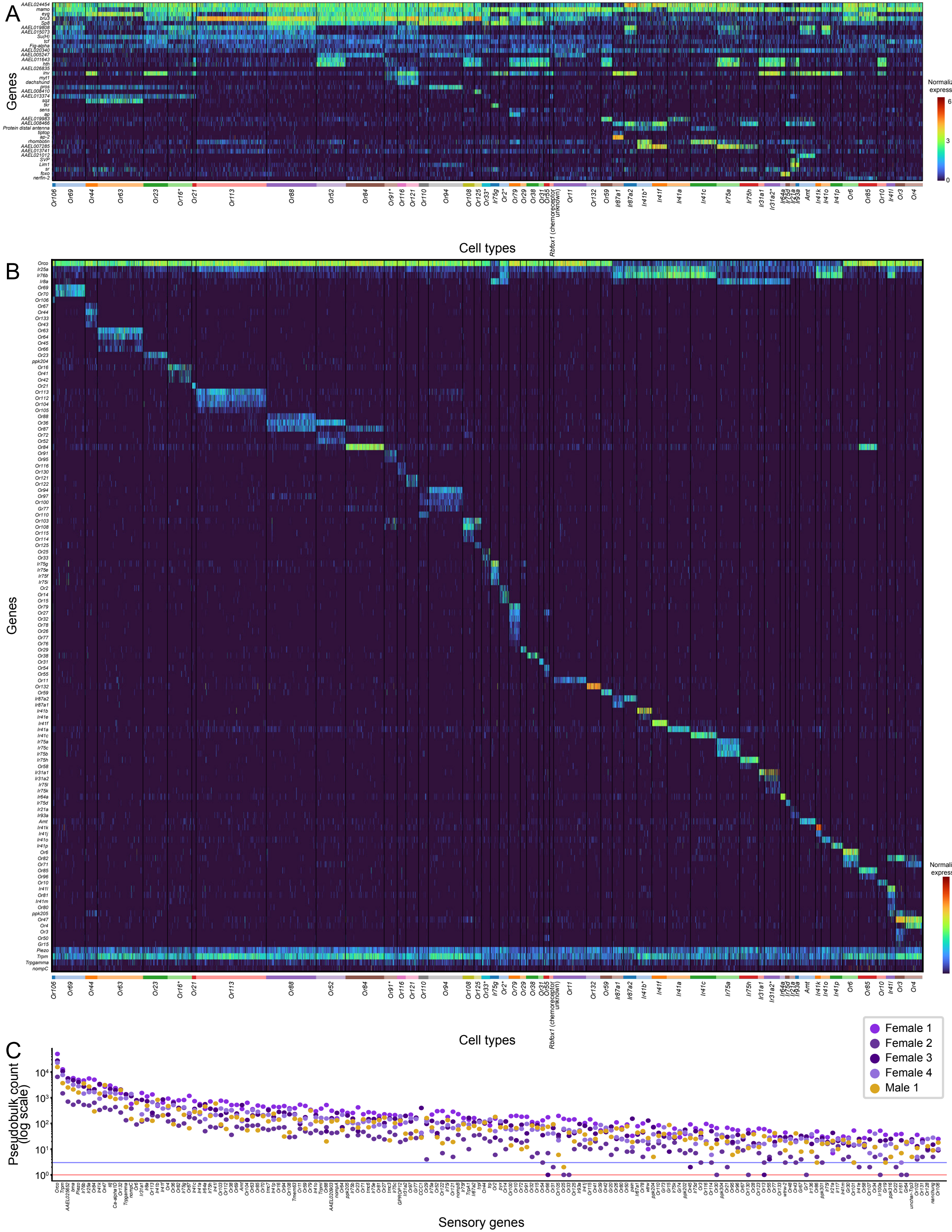
A
B
C
Genes
Cell types
Normalized expression
Sensory genes
Pseudobulk count (log scale)
Female 1
Female 2
Female 3
Female 4
Male 1
